# Supplementary material for: Self-organization of collective escape in pigeon flocks
Source: PLoS Comput Biol. 2022 Jan 10;18(1):e1009772. doi: 10.1371/journal.pcbi.1009772 (PMC8782486; doi:10.1371/journal.pcbi.1009772)
Supplement: S1 Table — Outliers (above the top 99% of the distributions) have been removed. Since in the real flocks individuals are still taking off at the beginning of the pursuit, we further removed the bottom 1% of the speed distributions of the empirical data. Our simulated data fall within the range of real flocks. (PDF) [file pcbi.1009772.s001.pdf]

Supporting Information

Self-organization of collective escape in pigeon flocks

Marina Papadopoulou [1], Hanno Hildenbrandt [1], Daniel W.E. Sankey [2], Steven J. Portugal [3], Charlotte K. Hemelrijk [1]

1 Groningen Institute for Evolutionary Life Sciences, University of Groningen, Groningen, The Netherlands
2 Centre for Ecology and Conservation, University of Exeter, Penryn, U.K.
3 Department of Biological Sciences, School of Life and Environmental Sciences, Royal Holloway University of London, Egham, U.K.

Supplementary Tables

S1 Table. Comparison of summary statistics of speed and nearest neighbor distance between 20 real and 20 simulated flocks. Outliers (above the top 99% of the distributions) have been removed. Since in the real flocks individuals are still taking off at the beginning of the pursuit, we further removed the bottom 1% of the speed distributions of the empirical data. Our simulated data fall within the range of real flocks.

Measurement	Type	Min.	1st	Median	Mean	3rd	Max
Speed (m/s)	Empirical data	12.22	13.99	15.61	15.71	16.77	21.26
		11.70	20.17	23.51	22.63	25.12	27.43
		11.19	14.56	16.13	16.02	17.37	20.07
		12.19	15.08	19.30	19.43	23.90	26.42
		14.52	19.61	21.29	20.85	22.86	24.46
		11.19	14.52	16.87	16.73	19.18	21.92
		14.84	17.60	22.14	21.28	24.66	26.60
		14.51	16.18	17.54	17.64	18.91	22.61
		10.29	15.67	17.96	17.38	19.48	21.16
		12.83	16.11	18.98	18.83	21.38	23.77
		12.94	14.60	16	15.95	16.90	20.07
		9.39	14.33	16.77	16.59	19.22	21.33
		12.08	15.71	16.83	16.75	18.13	20.45
		16.43	19.07	22.13	21.44	23.48	29.76
		10.49	15.63	17.01	17.38	19.43	23.58
		10.80	19.11	21.94	20.89	23.98	26.73
		10.84	15.41	17.26	16.56	18.39	20.61
		14.56	16.92	18.46	18.70	20.50	23.09
		13	15.24	17.97	17.51	19.70	21.96
		11.40	15.80	20.24	18.96	21.50	23.73
	Simulated data	13.95	19.30	20.01	20.02	20.58	24.60
		15.15	19.17	19.68	19.59	20.08	24.20
		14.70	18.49	19.15	19.06	19.69	23.21
		14.58	18.91	19.58	19.60	20.13	24.16

(To be continued)

Measurement	Type	Min.	1st	Median	Mean	3rd	Max
		14.15	18.17	18.90	18.74	19.40	22.74
		14.51	18.91	19.68	19.81	20.33	24.37
		14.04	19.18	19.81	19.85	20.41	24.61
		14.67	19.24	19.88	19.91	20.46	24.57
		14.58	18.64	19.35	19.27	19.89	23.62
		14.54	18.34	19.03	18.92	19.52	23.03
		14.93	18.82	19.57	19.44	20.02	23.26
		14.08	18.50	19.05	19.02	19.59	23.88
		14.59	18.25	18.97	18.86	19.50	22.50
		14.68	18.41	19.02	18.93	19.51	23.26
		14.78	18.75	19.42	19.37	19.90	23.43
		15.25	18.77	19.38	19.33	19.94	23.09
		14.76	18.31	19.04	18.93	19.52	23.38
		13.73	18.19	18.93	18.86	19.47	23.81
		15.01	19.04	19.71	19.68	20.31	23.88
		14.50	19.16	19.80	19.84	20.45	23.95
NND (m)	Empirical data	0	0.83	1.23	1.46	1.89	5.59
		0.14	1.03	1.72	1.88	2.54	6.38
		0	0.75	1.22	1.53	2	5.84
		0.13	0.91	1.56	1.92	2.41	10.78
		0	0.84	1.39	1.97	2.18	11.08
		0	0.75	1.33	1.72	2.23	7.11
		0	0.53	0.84	1.07	1.41	4.46
		0	0.73	1.24	1.63	2.06	9.17
		0	1.34	1.89	2.02	2.43	7.97
		0.11	1.10	1.63	2.06	2.64	10.33
		0.07	0.84	1.26	1.65	1.97	12.83
		0	0.96	1.72	1.84	2.49	7.59
		0	0.67	1.08	1.38	1.81	7.98
		0.07	1.19	1.90	2.23	2.75	11.14
		0	0.83	1.34	1.74	2.13	9.27
		0.14	0.90	1.53	2.13	2.48	17.90
		0	0.53	0.85	1.07	1.37	5.29
		0	0.77	1.30	1.60	2.12	5.94
		0	0.89	1.48	1.62	2.01	6.17
		0	0.57	0.92	1.25	1.50	8.78
	Simulated data	0	0.78	1.23	1.67	2.05	8.80
		0	0.88	1.23	1.41	1.70	5.73
		0	0.61	0.94	1.08	1.37	4.69
		0	0.65	1.06	1.71	1.80	11.87
		0.01	0.62	0.92	1.01	1.27	3.64
		0	0.68	1.19	3.21	3.04	31.99
		0	0.76	1.19	1.59	2.07	8
		0	0.79	1.23	1.70	2.01	11.49
		0.01	0.62	0.96	1.16	1.42	5.89
		0	0.60	0.91	1.06	1.32	5.25
		0	0.58	0.90	1.06	1.33	5.37
		0.02	0.68	1.02	1.18	1.44	6.93
		0.01	0.58	0.89	1.01	1.28	4.37
		0.01	0.62	0.93	1.05	1.33	4.52
		0	0.61	0.96	1.20	1.46	6.08
		0.01	0.59	0.91	1	1.29	3.88
		0.01	0.57	0.89	1.05	1.30	6.36
		0	0.59	0.89	1.06	1.29	7.96
		0	0.64	1.02	1.33	1.58	6.99
		0	0.76	1.21	1.63	1.99	8.82
