# Supplementary figures and images for: Self-organization of collective escape in pigeon flocks
Source: PLoS Comput Biol. 2022 Jan 10;18(1):e1009772. doi: 10.1371/journal.pcbi.1009772 (PMC8782486; doi:10.1371/journal.pcbi.1009772)

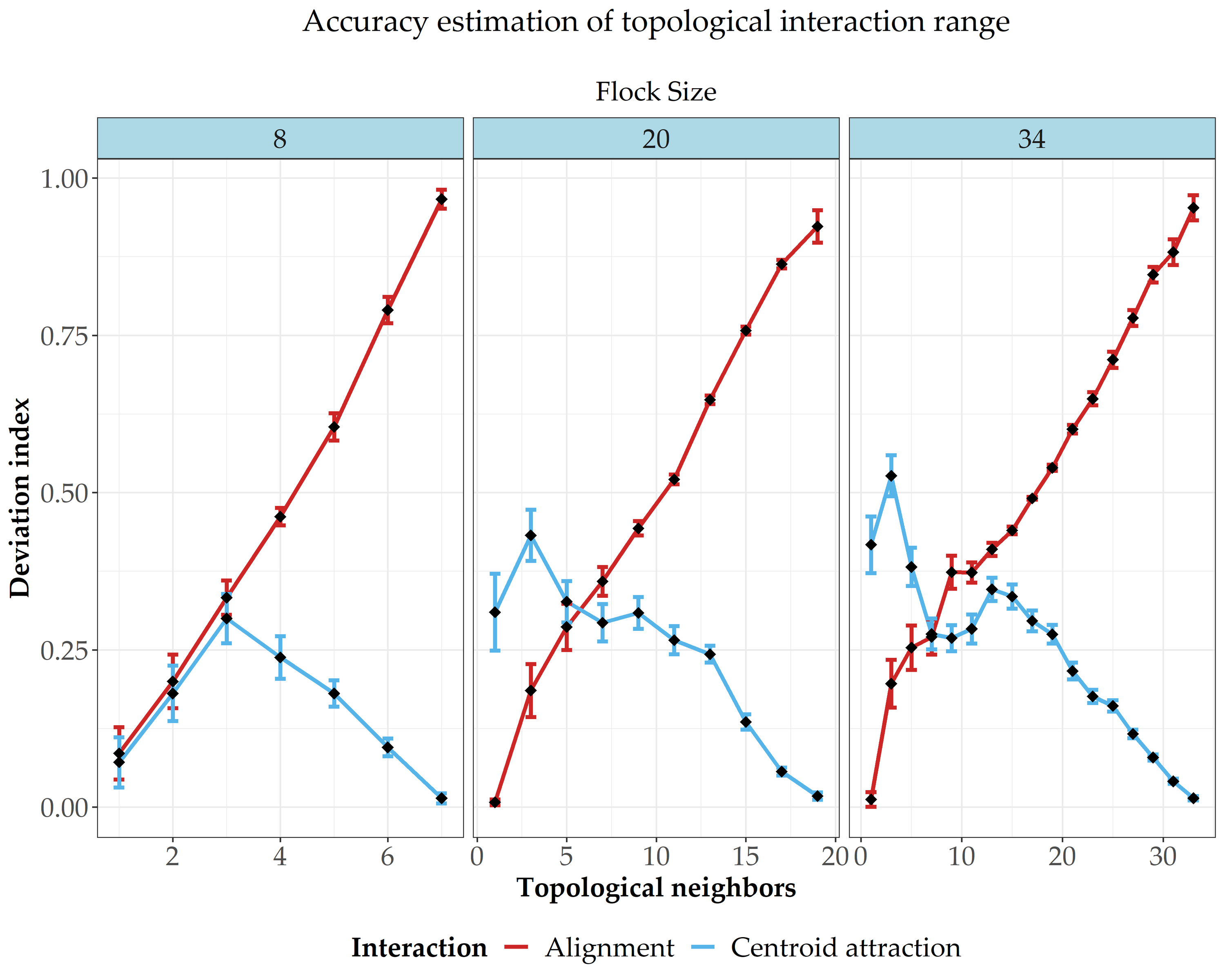

Supplement: S1 Fig — Their method is based on simple linear models between, on one hand, the turn that each individual performs during consecutive sampling points and, on the other hand, the turning angles for centroid-attraction and alignment. Angles based on all possible topological ranges for each flock size were tested. The linear model with the most explanatory power was thought to include the ‘real’ topological range. For the exact method description see [24]. Given that this method is not well established, we tested its performance on our simulated datasets. Specifically, we applied this method on data from simulations in which we vary separately the topological range for alignment and centroid-attraction (from 1 to all neighbors) for three flock sizes. We run 5 repetitions of each simulation with all unique combinations of topological range for alignment and centroid-attraction per flock size. The deviation index shows the deviation of the topological estimate of the linear-models method from the real value of topological range as parameterized in the model (shown on the x-axis), divided by the maximum possible deviation for each topological range and flock size (n-2, e.g. the maximum deviation for a flock of 8 individuals is 6 neighbors, when the true value is 7 and the estimate is 1 or vice-versa, giving a deviation index of 1). Values close to 0 show a good performance of the linear-model method. The method seems to lose accuracy when agents align with many topological neighbors and when they are attracted to the centroid of a few. Each point shows the mean deviation index of all simulations with the respective topological range and the error bars the standard error. (TIF) [file pcbi.1009772.s002.tif]

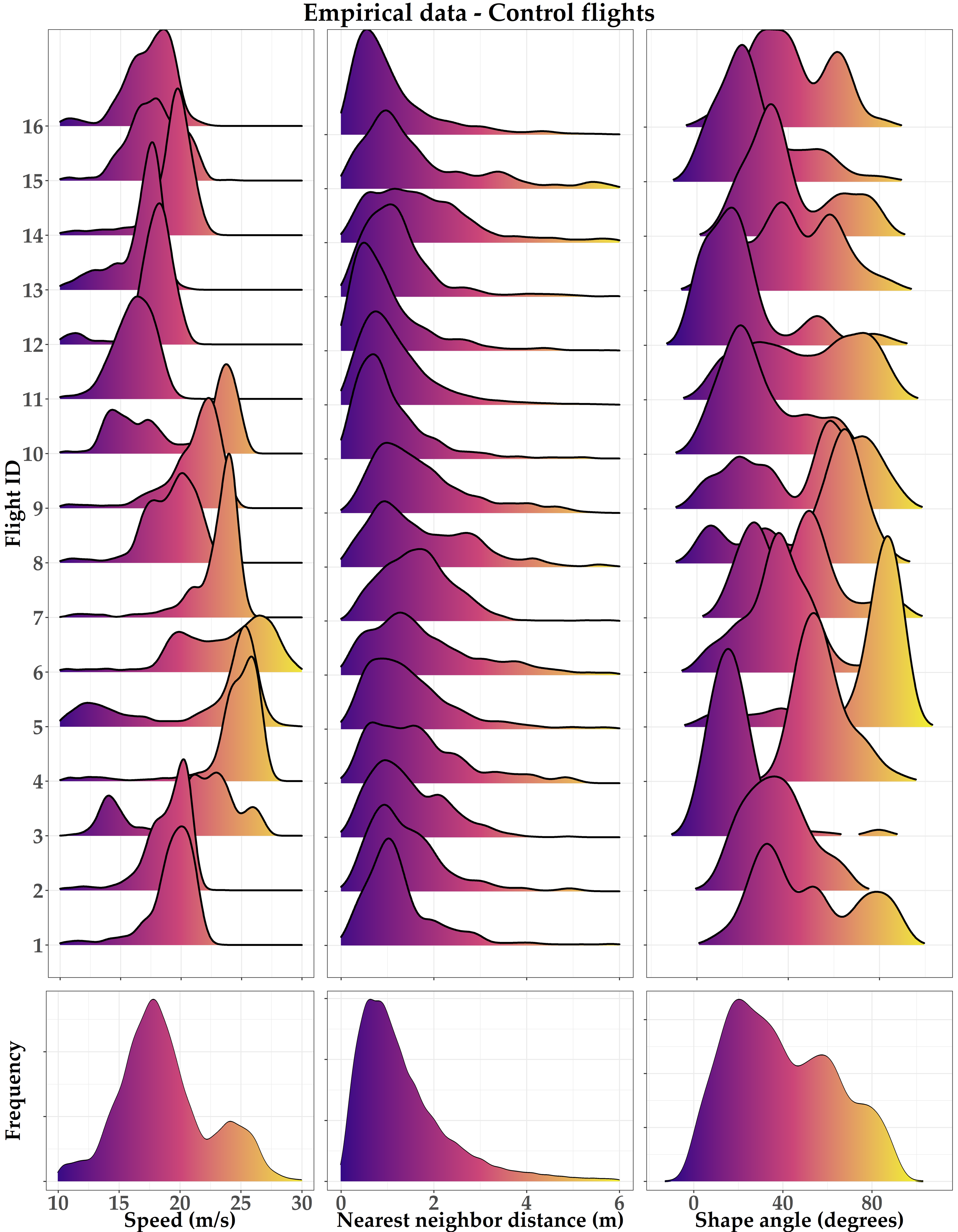

Supplement: S2 Fig — Each histogram shows the distribution of one flock during a control flight (based on the data of Sankey et al. (2021) [24]). The bottom row shows the overall distribution across flights. (TIF) [file pcbi.1009772.s003.tif]

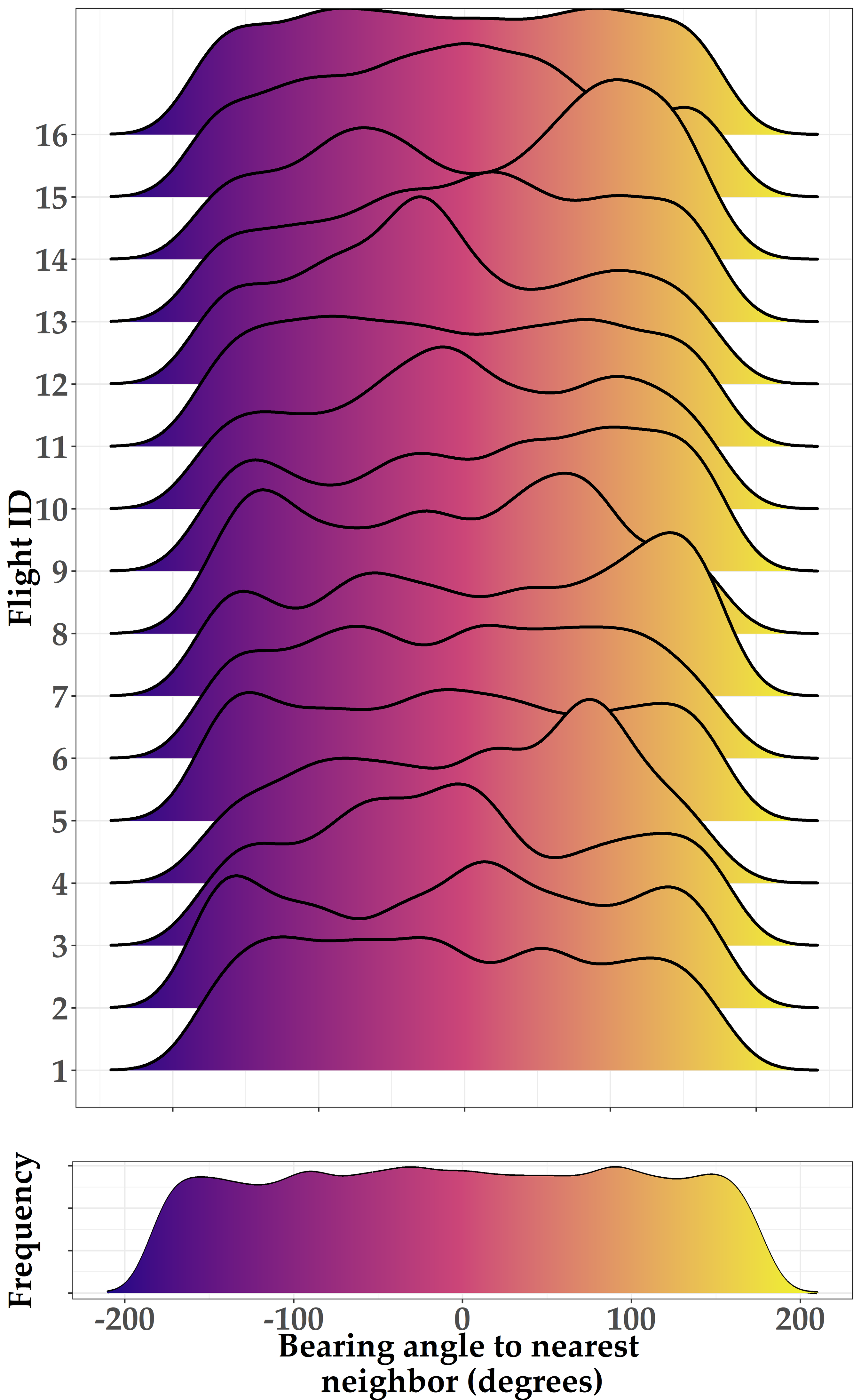

Supplement: S3 Fig — The overlapping histograms show the distribution of one flock during a control flight (based on the data of Sankey et al. (2021) [24]). The bottom row shows the overall distribution across flights. (TIF) [file pcbi.1009772.s004.tif]

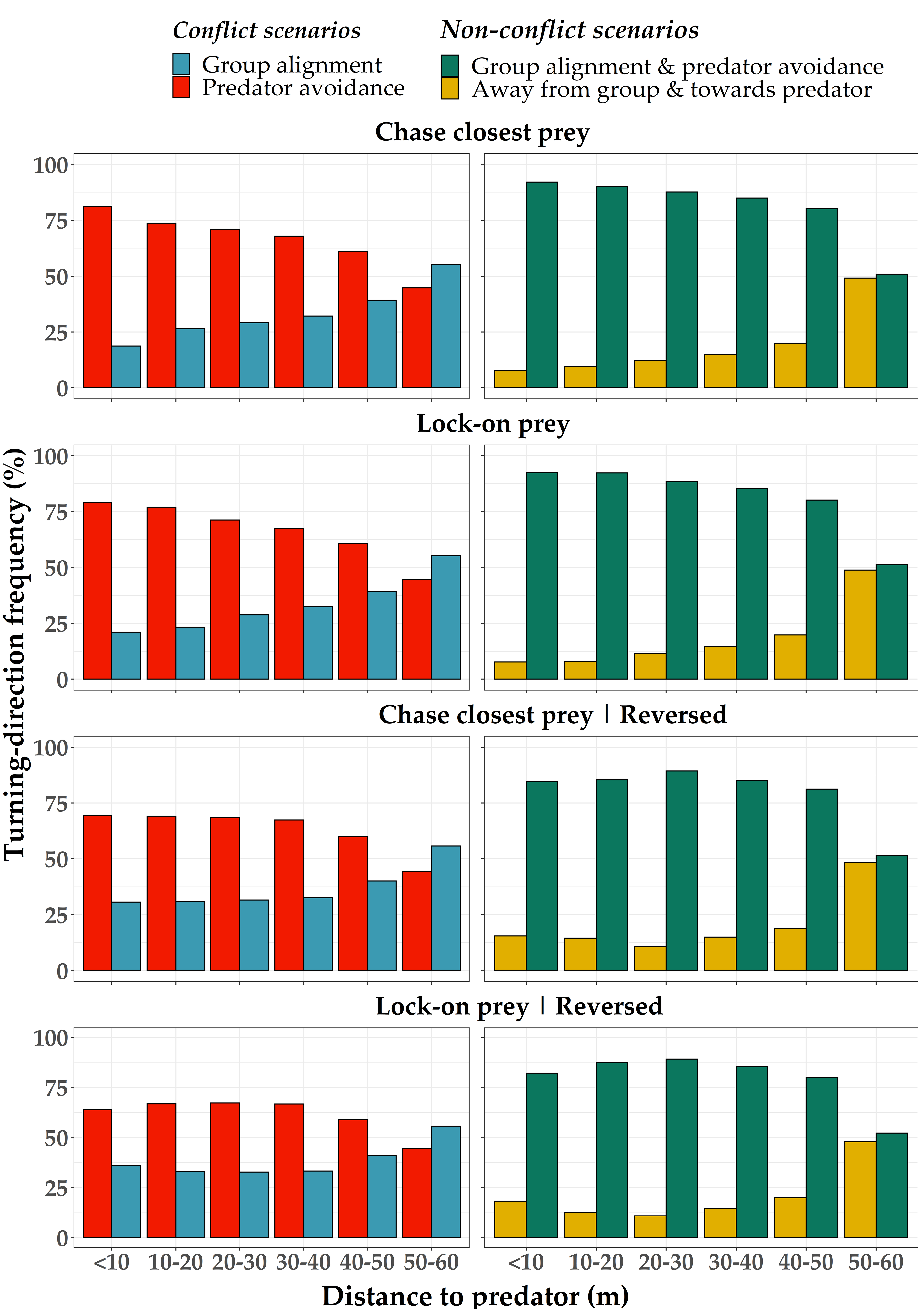

Supplement: S4 Fig — The default strategy is the ‘chase closest prey’. (TIF) [file pcbi.1009772.s005.tif]

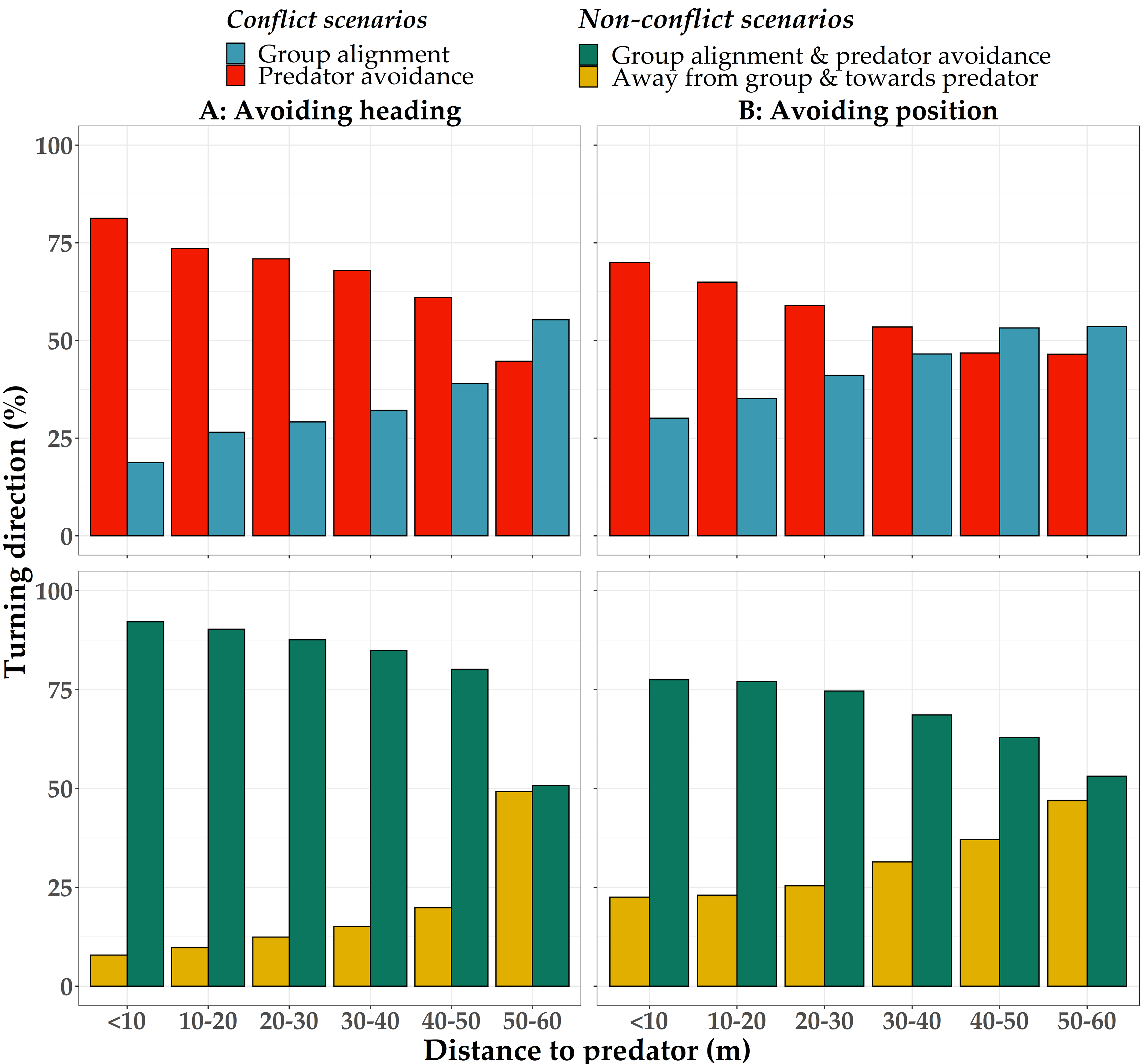

Supplement: S5 Fig — The default strategy, supported by the empirical data [24], is avoidance of the predator’s heading (A). The pattern of increased escape frequency of pigeon-agents at closer distance to the predator-agent holds for both strategies. (TIF) [file pcbi.1009772.s006.tif]
