# Supplementary material for: Self-organization of collective escape in pigeon flocks
Source: PLoS Comput Biol. 2022 Jan 10;18(1):e1009772. doi: 10.1371/journal.pcbi.1009772 (PMC8782486; doi:10.1371/journal.pcbi.1009772)
Supplement: Attachment — Submitted filename: ResponseLetter-Rev-SelfOrgPigeons-PLOSCompBio.pdf [file pcbi.1009772.s007.pdf]

19 October 2021

Dear Editors, Dr Fu and Dr Komarova,

Thank you for the opportunity to resubmit a revised version of our manuscript entitled: "Self-organization of collective escape in pigeon flocks" for consideration in PLOS Computational Biology. We highly appreciate the time that you and the three referees devoted to our work, and are thankful for the valuable feedback received through these reviews. We have incorporated changes that reflect the comments and suggestions provided by the reviewers. In detail, we revised our Abstract and Introduction, extended several parts of our Methods and Results, added a table and a figure in the supplementary (including newly performed simulations), and substantially expanded our Discussion. All changes are in red font in the revised manuscript. Please find below our point-by-point response to the reviewers (please note that line numbers refer to the revised version of the manuscript).

We look forward to hearing from you regarding our resubmission and to respond to any further questions and comments.

Sincerely,

On behalf of all authors, Marina Papadopoulou

Comments from Reviewer #1:

Major comment 1: The authors claim that the rule of predator-avoidance is independent of predator-prey distance. However, the analyses of the rule are actually based on the distance between predator-prey distance (e.g. Fig. 5). The closer the distance, the greater the difference in the direction from the predator. Yes, there is no direct distance as the input in the model, but the authors cannot reject the hypothesis that the direction change is related to the distance, which is clearly shown in the experimental data and simulation data. Moreover, there is a distance filter before applying this rule (e.g. dis<60), so there is at least a distance-based threshold for the rule. Overall, the distance is still a direct or indirect variable in the model.

Response: We apologize if it was not clear in our submitted text, but we wanted to say that having an individual rule through which individuals prioritize predator-avoidance when the predator is closer, is not necessary. Note that this rule was assumed in the initial explanation of the empirical pattern (Sankey et al. 2021). Our model shows that even if individuals do not consider their distance to the predator in their behavioral rule/intention, they still behave collectively as if they do. In other words, even though our behavioral rule is independent of distance, the individual behavior during collective escape is not. This is caused by the interactions among the individuals that cumulate into collective turning over time. In total, the distinction between the behavioral rule (rule of avoidance) and the actual individual and collective behavior (escape) is vital for understanding the process of self-organization we present here. Throughout our text we aim to distinguish between the two: the behavioral rule and the collective, emergent behavior. To further emphasize this, we have now revised our Abstract and Introduction, explicitly stated this difference in our Methods (lines 334-338) and revised our related discussion (lines 560-575). Concerning the distance filter, we included it because this is the range in which escape frequency is studied in the empirical experiment (we have also included this note in lines 292-293).

Major comment 2: It is not clear how did the author control the predator. There are several known strategies for predators, including anticipation (Mischiati et.al 2015 Nature) and proportional navigation guidance law (Brighton et.al 2017 PNAS). A direct pursue is not common in Nature. The last paper (Sankey et.al 2021 Current Biology) from these authors describes that the robot predator is controlled by a human. Commonly, human uses anticipation in the visual-based control. So, it would be reasonable to apply an anticipation-based algorithm for the predator.

Response: The reviewer raises an interesting point. However, the operator of the robot predator in the empirical study used only direct pursuit and approached the pigeons from behind (as described in Sankey et al. 2021). Therefore, this is the relevant attack procedure for studying in the computational model. Note that for the operator it is difficult to use an anticipation-based technique because: 1) he controlled the falcon by looking through the camera on the robot's head (not by looking at the flock from a distance) and 2) he had a short time-window to attack the flock at the location of departure. Thus, he could not anticipate the flock's motion and could only follow it. Furthermore, direct pursuit is a common hunting strategy of peregrine falcons on flocks (Zoratto et al. 2010 J. Avian Biol). We have now clarified this point in our Methods (lines 229-231) and included it in our Discussion (lines 666-677).

Major comment 3: The process of flocking after the initial turn of one agent is quite similar to the studies of phase transitions with two leaders (Couzin et.al 2005 Nature). In this paper, one "leader" is the agent who initialized the turning (predator avoidance), and the other "leader" is by the group (Group alignment). From Fig. 4, it is also clear that the phase transition is geometry (distance) based. "Group alignment" wins when the distance is larger, while "predator avoidance" wins when the distance is smaller. A compromise happens when the distance is moderate. This also reminds me of a recent paper by Sridhar et.al (2021 bioRxiv). It would be great to add a comparison of this phase transition in the experiments and simulations.

Response: This is indeed an interesting parallel; we thank the reviewer for their suggestion. We have however some concerns about the specific differences between our system and the mentioned studies. First, the 'directions' in our simulations (towards the flock or away from predator) are not global (but relative to each individual's heading), and second, we have no agents with a preferred direction that is constant throughout a pursuit. In other words, the 'leaders' are mentioned by the reviewer (predator avoidance and group alignment) are neither global entities nor constant over time. For instance, individuals that initialized the turning in one direction when the predator is further away, may actually turn towards the opposite direction when the predator is closer while the rest of the flock is turning towards the initial escape direction. The predator may change attack direction and the flock its direction of escape at any point. Thus, this differs from the predefined 'preferred direction' of leaders in the mentioned examples with phase transitions. To include this point in our manuscript, we have added a paragraph in our Discussion (lines 606-624)

Major comment 4: The analyses of Fig. 6 are similar to those complex contagions shown in fish school (Rosenthal et.al 2015 PNAS). The group behavior is not directly (fully) controlled by the initiator. They are affected by the neighbors who are affected by the initiator. Therefore, individuals are indirectly affected by the initiator. It would be nice to add a such discussion.

Response: We agree that this is a very interesting comparison to make and thank the reviewer for their suggestion. We have extended our Discussion to include it (lines 580-591).

Minor comment 1: Page5, line 161, the authors mentioned this \Phi_i in Fig. 1, but there is no such symbol in Fig. 1

Response: The reviewer must refer to the \Psi_i reference. We clearly see our mistake and source of confusion. We have revised the text accordingly (now lines 160-163).

Minor comment 2: Page8, line 244, why update asynchronously?

Response: This relates to our aim for our simulations to resemble real flocks, since birds update their information and reaction to their surroundings asynchronously; total synchrony of reaction is impossible in nature. Such asynchronous update has been used in previous computational models and shown to increase the noise of the system. We understand that this is not clear in the text and we have thus extended that section (lines 245-249).

Minor comment 3: Page10, Fig. 2 \ Theta_{a_j} in the figure should be \theta_c in the caption?

Response: This is indeed an inconsistency from our part, we apologize and thank the reviewer for noticing it. We have now made the symbol consistent across the figure, caption and text.

Minor comment 4: Why simulation in 2D, while experiments are in 3D. Add a discussion.

Response: We would like to thank the reviewer for this suggestion, we acknowledge that this is an important point missing. We have now extended our Discussion (lines 685-691) explaining the 3 main reasons behind our practice: a) to allow comparisons with the analysis of the empirical data by Sankey et al. 2021 that was done only using the x and y coordinates of the 3D data, and the fact that b) pigeon flocks are generally flat (little expansion in altitude), Yomosa et al. 2015 Plos ONE) and c) collective turns are often on a plane, even in 3-dimentional flocks of starlings (Attanasi et al. 2014 Nature Physics).

Comments from Reviewer #2:

Major comment 1: Because the proposed model is an agent-based framework, it includes a mass of parameters. The partial parameters are determined according to the empirical values in previous works and others are determined by visual testing. Does the values of these parameters (especially for these parameters determined by the visual testing) change largely with respect to the different flock sizes?

Response: No, our parameters are identical for all flock sizes. To clarify this, we have extended our methods accordingly (lines 142 and 265). Concerning the parameters, we took into account in our model, Sankey et al. 2021 did not find differences between large and small flocks. In our simulations, we chose flocks sizes identical to those in their experiment (Sankey et al. 2021). In general, these flock sizes are representative of the majority of previous work on pigeon flocks. Furthermore, we tuned many parameters in our model after Pettit et al. 2013 (J.R.S.Interface) who studied pairs of pigeons. By including their findings in our model we get simulated flocks that resemble empirical flocks with up to 34 flock members. Thus, there is evidence that these parameters are robust across group sizes. However, no data (to our knowledge) are available for very large flocks (>50 pigeons).

We have added a table in our Supplementary Material that shows the variation in speed and nearest neighbor distance across our simulated flocks, and the fact that this variation falls within the range of the empirical distributions (S1 Table). Note that even if small deviations in the parameters are present in real flocks due to flock size, their inclusion in the model will increase the complexity of our model without necessarily improving our understanding of the mechanism of collective escape we study (Grimm et al. 2005 Ecological Modelling, see also our addition to the Discussion, lines 682-684).

Major comment 2: Do the flock sizes dynamically evolve in the escaping process? If yes, how do the flock sizes changes with the evolving time? How does it statistically analyze the experimental results? In addition, the non-trivial comment is that "what is the important differences of the proposed model and conclusion between the current work and the work in reference [24]".

Response: No, flock sizes do not evolve in the model, since we follow the empirical study of the RobotFalcon [24]. Specifically, flock sizes stay constant, because we do not model the predator to `catch' prey and pigeon-agents do not die. We hope to improve clarity on this point by the revision in our Methods (lines 268-269). We think that studying pursuits from a real predator while allowing our population to evolve is a great potential extension of our model for future research (as we also state in our Discussion, line 695).

Concerning the important differences between our conclusions and those of the empirical study [24], we revised and extended the sections at the beginning of our Discussion where we address this point (lines 566-579). Specifically, our main point is that pigeons do not need to take their distance to the predator into account in order to increase their avoidance frequency as the predator gets closer. This is opposite to the conclusion of the work in reference [24] that pigeons are expected to prioritize predator avoidance over group alignment. Thus instead of individual decisions to turn to escape more as the predator gets closer, flock members increase their escape frequency through consensus over an escape direction and reinforcement during collective turning. Understanding this emergent pattern would not be possible without a computational model by the mere observation of the empirical data.

Comments from Reviewer #3:

Major comment 1: The authors performed simulations where the pigeon is modeled to sense only the predator heading, and not the predator position. These simulations showed an increased escape frequency when predator getting closer. Can the authors perform new simulations where the pigeon senses both the predator heading and predator position, or where pigeon only senses the predator position? Will these new simulations also show increased escape frequency? If so, the conclusion that pigeon senses only the predator heading may not be true.

Response: We thank the reviewer for raising such an interesting point, we agree that exploring the pattern under the different scenarios is of interest. According to the empirical study of Sankey et al. 2021 pigeons react mostly to the heading of the predator (and even turn a bit *towards* the predator's position); that's why we modelled the escape rule of avoiding the heading. To examine your suggestion, we have run new simulations for individuals avoiding either the predators' position or both its position and heading. We analyzed the data like in Figure 4 of the manuscript and compared the new results with our original simulations in which the heading of the predator is taken into

account (Figure R1). The pattern holds (a bit less strongly) for position avoidance, but it is less clear for the case where prey take both heading and position into account. To understand better the mechanism across this alternative scenario will require in depth theoretical investigation that falls out of the scope of this paper, given that we aim to keep our simulations as close as possible to the experiment with real pigeons (where only heading avoidance is suggested). We added the plots from Figure R1 A & B in our Supplementary Material (S5 Fig) and raised the point of alternative strategies of predator avoidance in our Discussion (lines 653-664).

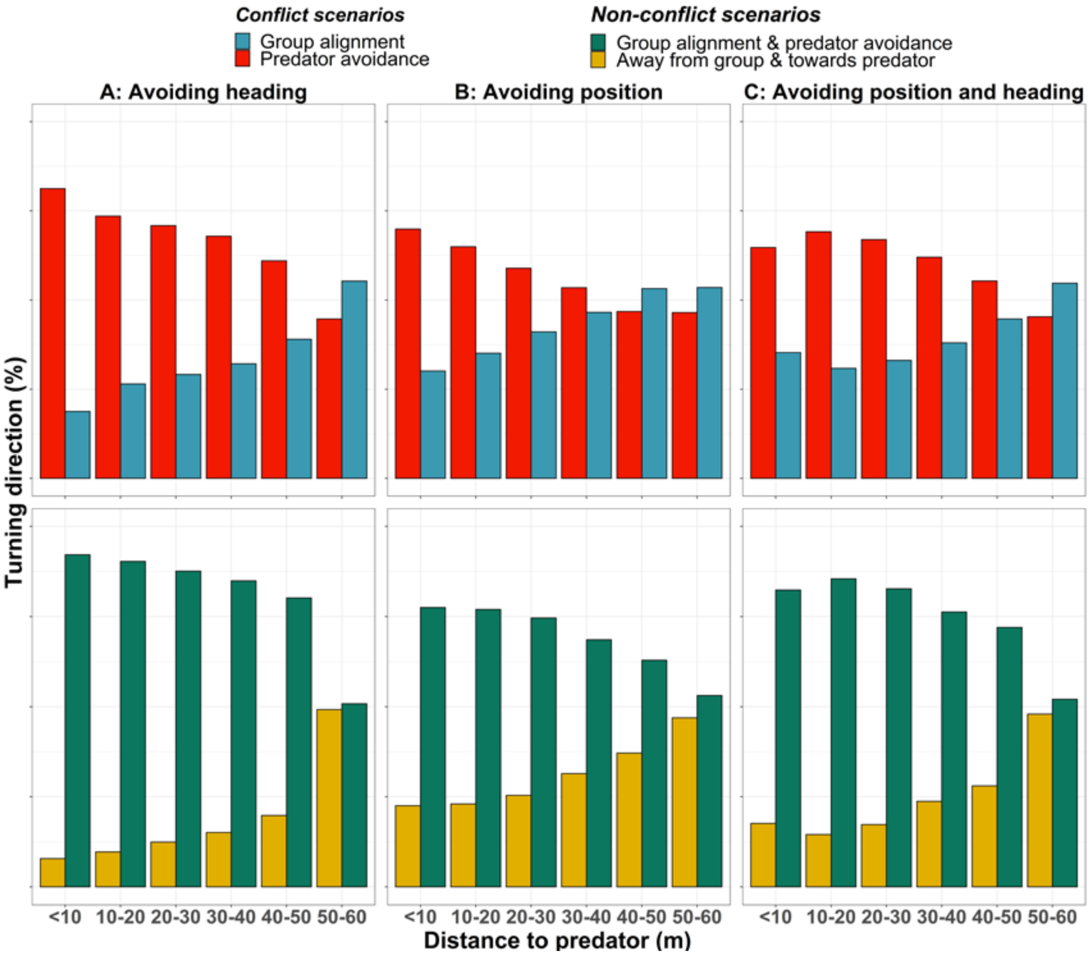

Figure R1. Escape frequency with avoidance strategies based on: the predator's heading (A), position (B), or both (C).

Major comment 2: Although the authors showed a good agreement between the simulation results and experimental data in Figure 3, this comparison is only for one of the 43 flocks collected in the experiments. The authors also realized that there is a large variability in the measurement of real flocks. Thus, it is might be doubt whether the conclusions draw from this simulation can be applied to other pigeon flocks, e.g., flocks with larger number of birds.

Response: We would like to thank the reviewer for pointing out that our comparison with only 1 flock might be a source of doubt for the reader. The distributions we showed are representative of the majority of pigeon flocks in the empirical data. To give a more complete picture, we have added a table in our supplementary with the summary statistics of speed and nearest neighbor distance from

20 randomly selected empirical and simulated flocks (S1 Table). The variation among the flocks we simulated falls within the range of the empirical data. In addition, note that we aim for a minimal representation, a caricature, of a real flock; trying to capture more variability seen in the empirical data is not needed (Grimm et al. 2005 Ecological Modelling). We aim to keep the right balance between simplicity of the model and the complexity in the empirical data.

Nevertheless, to further test that our conclusions are robust across flocks with different characteristics within the range of the empirical data, we run a sensitivity analysis varying parameters that affect the most the distributions of nearest neighbor distance (minimum separation distance) and speed (mean cruise speed). We validate that our main results are robust across all simulation sets (Figure R2). Concerning flocks with larger number of birds, our simulations only concern the group sizes of the empirical study. We do not know whether our study pattern holds in larger flocks of pigeons in nature. Thus, we think that simulations with larger flock sizes are out of the scope of this manuscript.

In sum, we have introduced the additional comparison with empirical data in the caption of Figure 3 and our Results (line 401), and added a discussion about the similarity of our model to real flock of pigeons (lines 683-685).

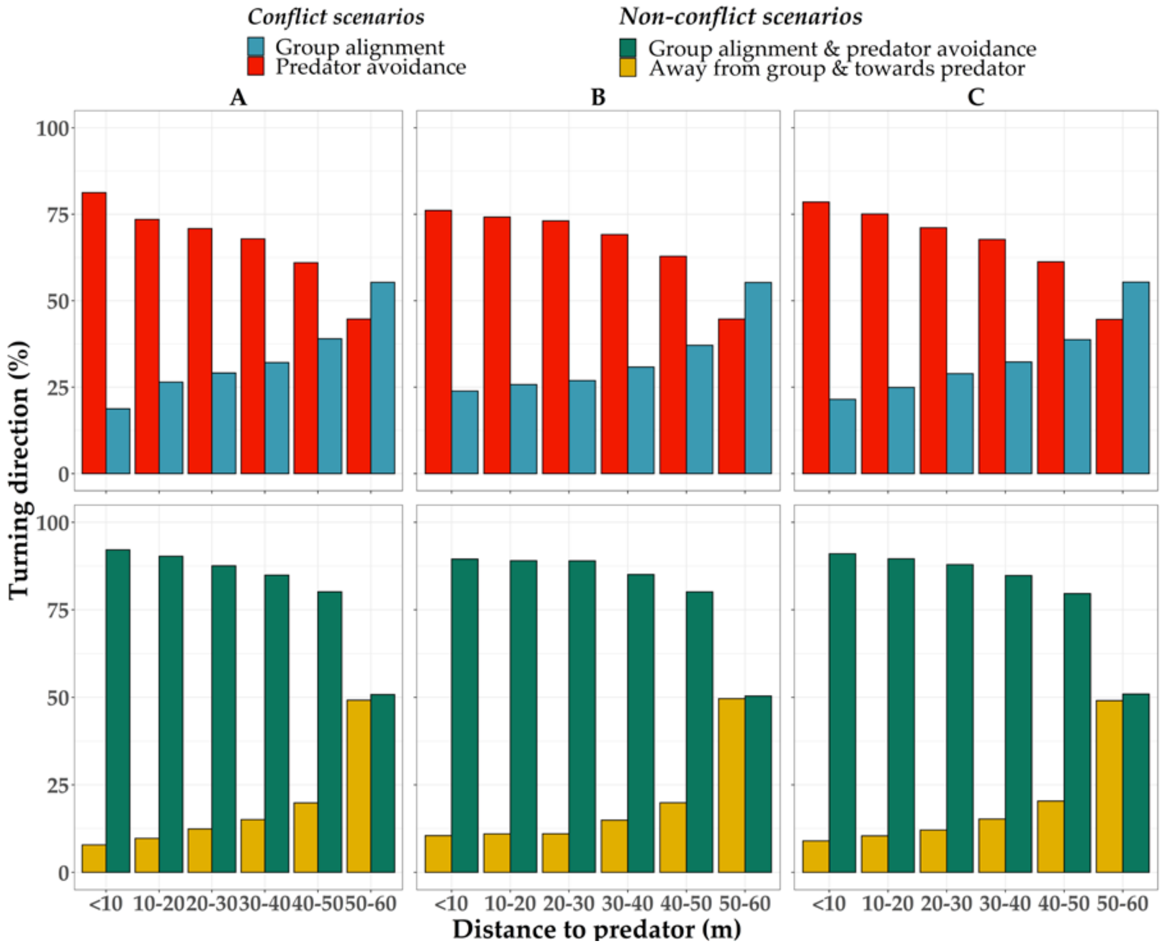

Figure R2. Escape frequency across different parameter sets. A: our main results presented in the manuscript (Figure 4B), B&C: flocks with smaller and larger values for minimum separation distance relative to our default parameters, C: faster flocks relative to the simulated data we present in our manuscript.

Major comment 3: The mechanism why the escape frequency increases when the predator getting closer is not well explained. Figure 5 shows several parameters which scale with the predator-prey distance. But it is not very clear why these parameters depends on the distance. The authors explained them through reinforcement. Why reinforcement only plays a role when the predator gets closer, not for predator moves apart?

Response: We thank the reviewer for pointing out this lack of clarity. To avoid confusion with the parameters in our model we will refer to measurements in Figure 5 as *variables*. We indeed extended our explanation of the scaling of these variables with the distance of prey to the predator in our Results (lines 433-450, 466-467, 555-557). Specifically, these variables do not depend on distance to the predator, but rather capture the state of the flock as the predator gets closer. For instance, the angle between the heading of prey and predator shows that (a) the flock is turning away from the predator as its distance to the predator decreases, and that (b) at close distance to the predator this angle is the largest, meaning that the predator intersects the flock from the side (relative to the flock's heading). The frequency of change in the escape direction is linked to the collective turn that is taking place. The larger the angle between the heading of the flock and the predator, the lower the likelihood that small deviations in the heading of a flock member will change the fact that its heading is to the left or to the right of the predator's. Since the relative direction of the heading of the predator to that of the flock members controls the individuals' escape direction, the larger the flock's angle with the heading of the predator is, the more stable the flock's escape direction will be. Thus, when very close to the predator, all flock members have reached consensus regarding which direction to escape from it. In sum, the measurements in Figure 5 reflect how the flock members reach consensus on which direction they should turn to avoid the predator, resulting in the increase in escape frequency of flock members during the attack. In the sub-set of simulations where the predator moves away from the flock, reinforcement cannot take place because the escape direction of each flock member changes often and the flock doesn't have enough time to reach consensus for turning collectively. We hope our addition to the manuscript increases the clarity of the mechanism of reinforcement.

Major comment 4: It seems like the escape mechanism described in Figures 6 and 7 are only true for one particular case, where there are two turns in an escape and the predator changes the direction of attack when getting closer to the flock. If the predator use a different attack strategy or attack from a different direction, will the flocks still use the same escape mechanism?

Response: We thank the reviewer for their question. The example in Figures 6 and 7 is a representative case of the emergent behavior of the flock and predator in our model. The change of attack direction comes from the fact that the predator overshoots, an effect that is expected when prey avoids a predator's heading as shown in a theoretical approach by Howland 1974 J.Theo.Biol. (see also the discussion of Sankey et al. 2021 Current Biology on the matter). The direction from which the predator attacks the flocks varies in our simulations (is not a direct input of the model). We now clarify these points in our Results (lines 473-476). We think that even though testing a variety of attack strategies is an interesting question, it is out of the scope of this manuscript, because it was not done in the empirical study. We have further incorporated this point in our Discussion (lines 659-662, 666-678).

Minor comment 1: Figure 1: The force of predator avoidance should be re-plotted such that it is perpendicular to the predator's heading.

Response: Actually the force of predator avoidance in the model is perpendicular to the pigeon-agents' heading (as plotted), representing just a turning direction away from the predator. We notice the source of confusion in the text and have revised it accordingly in the caption of Figure 1.

Minor comment 2: Line 193: The speed-attraction force in the main text is denoted as phi^cs, while it is denoted as phi^ca in Figure 1. The symbols should be consistent.
Minor comment 3: Line 309: The symbols are not consistent to Figure 2. The average heading of flock is denoted as rho_c in the main text, but rho_a in the figure. The angle between the flock heading and the individual heading is sometime denoted as theta_a, and sometime as theta_c.

Response (comments 2 & 3): We thank the reviewer for noticing and we apologize for this inconsistency. We have now made all symbol consistent across the text, captions and in the Figures 1 & 2.

Minor comment 4: Line 309: The authors distinguish between toward the flock and away from the flock by the sign of theta_a (the angle between flock heading and the individual heading). It is not clear to me how the sign of an angle is defined. What is the meaning for a negative angle?
Minor comment 5: Line 332, similarly, it is unclear how the sign of an angle is defined in Equation 7.

Response (comments 4 & 5): We indeed noticed that our text lacked clarity concerning the handling of signs. We thank the reviewer for pointing it out. We have now added the definition of the sign function (now eq. 6) and clarification on the range of our angles in the Methods (lines 315-321).

Minor comment 6: Line 320, (1b, 2b) should be corrected into (1b, 2a).

Response: We thank the reviewer for noticing, we have corrected the typo (now line 334).

Minor comment 7: Figure 3 C1 & C2, why the density is highest at a distance close to 0? I expected the highest density at the nearest neighbor distance of 1.3 m.

Response: Actually the highest density is expected to be at the mode of the distribution (approx. at 0.4 m). Since the distribution of nearest neighbor distance is skewed to the left, both the mode and median are lower than the mean. We understand that it is an easy misconception to make and we have thus clarified it in the caption of Figure 3.
