# Supplementary material for: Self-organization of collective escape in pigeon flocks
Source: PLoS Comput Biol. 2022 Jan 10;18(1):e1009772. doi: 10.1371/journal.pcbi.1009772 (PMC8782486; doi:10.1371/journal.pcbi.1009772)
Supplement: Attachment — Submitted filename: ResponseLetter2-Rev-SelfOrgPigeons-PLOSCompBio.pdf [file pcbi.1009772.s008.pdf]

16 December 2021

Dear Editors, Dr Fu and Dr Komarova,

Thank you for the positive reaction to our resubmitted manuscript entitled: "Self-organization of collective escape in pigeon flocks" and your consideration for accepting it for publication in PLOS Computational Biology.

We would further like to thank reviewer #1 for the additional valuable feedback. We have revised our Discussion to reflect their comments and suggestions. All changes are in red font in the revised manuscript. Please find below our point-by-point response to the reviewer (please note that line numbers refer to the latest revised version of the manuscript).

We look forward to hearing from you regarding our resubmission.

Sincerely,

On behalf of all authors, Marina Papadopoulou

Comments from Reviewer #1:

Major comment 1: The authors mentioned the robot falcon is controlled by an operator with a direct pursuit strategy. This is different from the falcon in nature (e.g., Brigthon et al. 2017 PNAS). One can naturally ask if the pigeons think the robot falcon is a predator? I think this is difficult to answer without behavior data of pigeons reacting to live falcons and robots. Without these data (even previous paper is accepted in Current Biology under the same descriptions), there might be many other hypotheses to explain these behaviors, e.g., obstacle avoidance. It would be great to add a bit of discussion in this direction.

Response: We thank the reviewer for raising an important question. Within our funded project, we have conducted studies to examine whether birds react to the RobotFalcon in a similar way as to a real predator (peregrine falcon) (of course we will never know what birds think). We show that the flocks' reactions to the RobotFalcon versus the real falcon are qualitatively the same (in terms of patterns of collective escape and frequency of reacting by several patterns) [1] and that they react less and differently to attacks by a common drone (Storms *et al.* in prep, [25] in the manuscript). Thus, the attack strategy of the RobotFalcon is similar to that of a falcon in nature (e.g. Zoratto et al. 2010 J. Avian Biol) and bird flocks react to it as to a real peregrine falcon. We believe this is the case with flocks of pigeons as well. If the reviewer finds it necessary, we are happy to confidentially share our unpublished results with him. We revised our discussion to include this information (lines 673-677).

[1] Rolf F. Storms, Claudio Carere, Robert Musters, Simon Verhulst & Charlotte K. Hemelrijk. *Patterns of collective escape of bird flocks under predation by a RobotFalcon.* ASAB Winter Meeting 2021, Oral Presentation and manuscript in preparation.

Major comment 2: Individual or collective decision-making under different options does not necessarily need leaders or those special individuals having information (see Sridhar et al., 2021 BioRixv). The system in this study exactly follows the logic described in the BioRixv paper, where the decision emerges regardless of distance sensing. In this paper, two options for the individuals are avoiding robots or flying together with other neighbors. How the individuals choose one of the options is the main question answered in this study. This question is exactly the same question raised in the BioRixv paper.

Response:

Indeed, in our work, the decision to turn away from the predator (option 1) instead of towards the group (option 2) emerges regardless of sensing the distance to the predator. However, in our study, individuals will always choose between the two options, averaging across both is not possible. Additionally, the group increases its selection of always the same option (collectively always turning away from the predator as the predator gets closer) and decreases the other (turning towards the flock) continuously (without a clear switching point). This is not the case in Sridhar et al. 2021, where individuals may choose to move in between the two options, and the group can potentially go towards any of the two. Another difference is that the two options in our system relate to the relative headings of individuals in the flock and the heading of the predator which continuously change during a pursuit, whereas in Sridhar et al. the two options are fixed in direction (or moving positions relatively fixed to each other at stable distance) and global in space. Based on this, an individual close to a virtual fish 1 cannot instantaneously move to be close to virtual fish 2 during a single time step, while in our model an individual can still, for instance, switch to option 1 after consecutively selecting option 2 (instantaneously turn towards the predator to stay with the group when the predator is close). Thus, we think that even though both papers concern collective decision making that is independent of sensing distance (between group members and leading virtual fish or the predator), the mechanisms in the two papers differ and therefore, emphasizing phase-transitions as presented in Sridhar et al. falls outside the scope of our manuscript. We revised our Discussion to improve clarity and reflect this discussion (lines 607-624).
